# Correntropy induced loss based sparse robust graph regularized extreme learning machine for cancer classification

**DOI:** 10.1186/s12859-020-03790-1

**Published:** 2020-10-07

**Authors:** Liang-Rui Ren, Ying-Lian Gao, Jin-Xing Liu, Junliang Shang, Chun-Hou Zheng

**Affiliations:** 1 School of Computer Science, Qufu Normal University, Rizhao, 276826 China; 2 Qufu Normal University Library, Qufu Normal University, Rizhao, 276826 China; 3 College of Computer Science and Technology, Anhui University, Hefei, 230601 China

**Keywords:** Extreme learning machine, Correntropy induced loss, Supervised learning, Bioinformatics

## Abstract

**Background:**

As a machine learning method with high performance and excellent generalization ability, extreme learning machine (ELM) is gaining popularity in various studies. Various ELM-based methods for different fields have been proposed. However, the robustness to noise and outliers is always the main problem affecting the performance of ELM.

**Results:**

In this paper, an integrated method named correntropy induced loss based sparse robust graph regularized extreme learning machine (CSRGELM) is proposed. The introduction of correntropy induced loss improves the robustness of ELM and weakens the negative effects of noise and outliers. By using the *L*_2,1_-norm to constrain the output weight matrix, we tend to obtain a sparse output weight matrix to construct a simpler single hidden layer feedforward neural network model. By introducing the graph regularization to preserve the local structural information of the data, the classification performance of the new method is further improved. Besides, we design an iterative optimization method based on the idea of half quadratic optimization to solve the non-convex problem of CSRGELM.

**Conclusions:**

The classification results on the benchmark dataset show that CSRGELM can obtain better classification results compared with other methods. More importantly, we also apply the new method to the classification problems of cancer samples and get a good classification effect.

## Background

Universal approximation capability plays a crucial role in settling regression and classification problems. Because of this ability, the single hidden layer feedforward neural network has always been the focus and hotspot of researches [1]. As a method to train the SLFNs [2], extreme learning machine (ELM) [3–8] has attracted the attention of researchers in recent decades [9]. Different from traditional neural network models, such as the backpropagation (BP) algorithm [10, 11], the training process of ELM is implemented in one step rather than iteratively [12]. In the original ELM, the first step is to randomly initialize an input weight matrix ${\mathbf{A}}$ and remain fixed throughout the process. Then, by using a nonlinear piecewise continuous activation function ${\text{g}} \left( x \right)$, the data of the input layer is mapped into the feature space of the ELM, and a hidden layer output matrix ${\mathbf{H}} = \left[ {{\mathbf{h}}\left( {{\mathbf{x}}_{{\mathbf{1}}} } \right),{\mathbf{h}}\left( {{\mathbf{x}}_{{\mathbf{2}}} } \right), \ldots ,{\mathbf{h}}\left( {{\mathbf{x}}_{{\mathbf{N}}} } \right)} \right]^{T}$ is obtained. Finally, by solving a ridge regression problem [13], the output weights ${{\varvec{\upbeta}}} = \left[ {{{\varvec{\upbeta}}}_{1} , \, {{\varvec{\upbeta}}}_{2} , \ldots , \, {{\varvec{\upbeta}}}_{L} } \right]^{T}$ connecting with the hidden layer and the output layer can be determined [14]. Since there is no need to iteratively solve the output weight matrix, compared with the traditional backpropagation algorithm, ELM can achieve better generalization performance at a faster speed [2, 3, 7]. Because of the advantages of simple theories, high efficiency, and low manual intervention, ELM has been used as a tool for various applications, such as image classification [15, 16], label learning [17], image quality assessment [18], traffic sign recognition [19], and so on.

Although it has been widely used, the robustness and sparseness of the ELM algorithm are still the hot topic. Huang et al. proposed RELM in [5] and in their method, $L_{2}$-norm was introduced to simultaneously constrain the loss function and the output weight matrix. Their experimental results provided that RELM was better than the original ELM. However, the square loss based on $L_{2}$-norm will amplify the negative impact of noise and outliers, and lead to inaccurate results. In [9], Li et al. introduced the *L*_2,1_-norm into ELM as a loss function and the regularization constraint. Hence, a new method named LR21ELM is proposed. The classification results showed that the robustness of the *L*_2,1_-norm was significantly better than the $L_{2}$-norm.

**Fig. 1:**
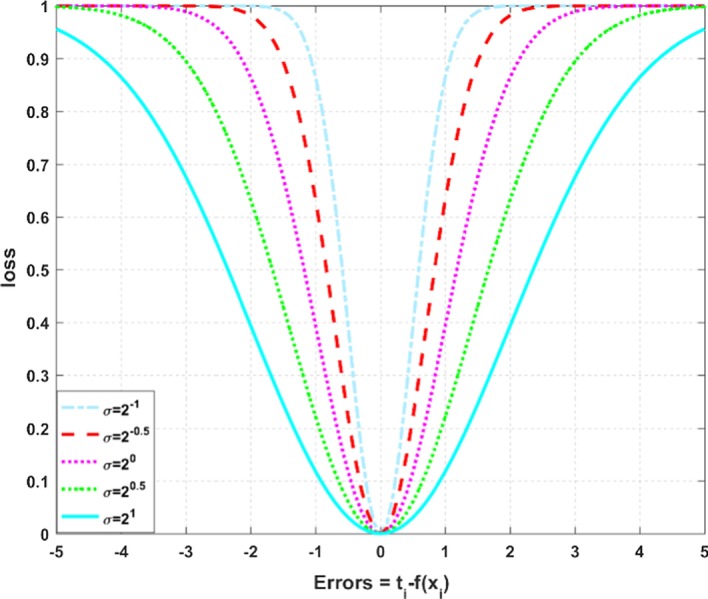
Correntropy induced loss with different kernel bandwidths

As a local similarity measure, correntropy is proposed based on the information theory and the kernel method [20]. Through a nonlinear feature mapping, correntropy can project the data from the input space into the feature space. It also computes the $L_{2}$-norm distance and defines a correntropy induced metric (CIM) in the feature space [21]. The correntropy induced loss [22] is defined as ${\text{C}} \left( {{\mathbf{t}}_{i} ,{\text{f}} \left( {{\mathbf{x}}_{i} } \right)} \right) = 1 - \exp \left( { - {{\left( {{\mathbf{t}}_{i} - {\text{f}} \left( {{\mathbf{x}}_{i} } \right)} \right)^{2} } \mathord{\left/ {\vphantom {{\left( {{\mathbf{t}}_{i} - {\text{f}} \left( {{\mathbf{x}}_{i} } \right)} \right)^{2} } {2\sigma^{2} }}} \right. \kern-\nulldelimiterspace} {2\sigma^{2} }}} \right),$ where ${\mathbf{t}}_{i}$ is the target vector, ${\text{f}} \left( {{\mathbf{x}}_{i} } \right)$ is the prediction matrix and $\sigma$ is the kernel bandwidth. Figure 1 depicts the correntropy induced loss function for different kernel bandwidths within the same error range. We can observe that correntropy induced loss is a non-convex, bounded, and robust loss function [23].

The robustness of correntropy to noise and outliers has been proved theoretically and experimentally. Ren et al. [21] integrated the correntropy loss and hinge loss (CH-loss) into ELM and proposed a robust extreme learning machine with the CH-loss (CHELM). They verified the robustness of the method at different noise levels. The results showed that correntropy loss could effectively reduce the influence of noise on classification results. In [24], Zhao et al. proposed the C-loss based ELM (CELM) and applied their method to estimate the power of small-scale turbojet engines. Chen et al. [25] introduced the correntropy loss to the multilayer ELM and proposed a robust multilayer ELM auto-encoder. The results showed that the feature extraction ability of the method was improved with the improvement of robustness.

In this paper, by integrating the correntropy induced loss into the ELM instead of the original $L_{2}$-norm, an integrated model named correntropy induced loss based sparse robust graph regularized extreme learning machine (CSRGELM) is proposed. Different from the traditional ELM, we use *L*_2,1_-norm instead of $L_{2}$-norm to constrain the output weight matrix to reduce the complexity of the neural network model. Moreover, the graph regularization is integrated into our method so that the neural network model can learn local structural information between data. This paper mainly makes the following research:

1. A new correntropy induced loss based sparse robust graph regularized extreme learning machine is proposed. Compared with the original ELM, the introduction of correntropy induced loss can improve the robustness. And the *L*_2,1_-norm is used as a sparse constraint to regularize the output weight matrix ${{\varvec{\upbeta}}}$, which can reduce the complexity of the model. To fully preserve the manifold structure information between the original data, the graph regularization is introduced into our method.
2. Based on the theory of [26], we design an iterative optimization method to cope with the non-convex problem of CSRGELM. The convergence and the computational time complexity of the new method are proved, respectively. We also design some experiments to prove the robustness of the method. It is observed that the robustness and classification ability of CSRGELM is better than that of ELM based on the traditional $L_{2}$-norm loss function. Compared to other robust ELMs, CSRGELM can also achieve competitive results.
3. We first perform the classification experiments on five benchmark datasets and evaluate the performance of CSRGELM through multiple evaluation measures. The results show that in most datasets, the classification results of CSRGELM are superior to other methods.
4. The new method is applied to the cancer sample classification problems of integrated TCGA datasets. Whether on integrated binary datasets or integrated multi-class classification datasets, the classification performance of CSRGELM is superior to other methods. The experimental results prove that CSRGELM can be a powerful tool for studying biological omics data.

## Results

Firstly, five benchmark datasets are used to evaluate the classification performance of RELM, *L*_2,1_-RFELM, LR21ELM, CELM [24], and CSRGELM. And then, CSRGELM is applied to the cancer sample classification tasks of the TCGA integrated datasets. In the experiments, the sigmoid function is chosen as the activation function. The evaluation criteria for testing classification performance are commonly used measures: Accuracy (Acc); Precision (Pre); Recall; F-measure (F-mea). Next, we will introduce the content of the experiment in detail.

### Evaluation criteria

According to the Table 1, the definition of each measure are as follows:

1 $$Acc = \frac{TP + TN}{{TP + FN + FP + TN}},$$

2 $$Pre = \frac{TP}{{TP + FP}},$$

3 $$Recall = \frac{TP}{{TP + FN}},$$

4 $$F{-}mea = \frac{2 \times Pre \times Recall}{{Pre + Recall}}.$$

**Table 1:** Classification results confusion matrix

The true situation	The predicted situation	
	Positive	Negative
Positive	True positive (TP)	False negative (FN)
Negative	False positive (FP)	True negative (TN)

For a multi-class dataset, we use one of the classes as the positive class and the remaining as the negative class to compute the accuracy, precision, recall, and F-measure. Finally, the average of every measure for all classes is obtained. All methods are conducted in MATLAB R2016a with 64 GB of memory and 3.60-GHz computer.

### Datasets

We use five popular benchmark datasets to test the classification performance, and every dataset has been widely applied in supervised problems [13, 27–29].

- Iris: Taken from the UCI database (https://archive.ics.uci.edu/ml/index.php), Iris is a multi-class classification dataset with 150 samples and 4 features, which is already widely used in unsupervised learning [30, 31] and supervised learning [5].
- COIL20: As a multi-class classification image dataset, the Columbia object image library is often used as a benchmark dataset to test the performance of machine learning methods. With 1024 features, it has 1440 samples, all of which are grayscale images of 20 different objects.
- USPST: As a subset of the popular handwritten digital recognition dataset USPS, USPST is the testing set of USPS. And it has 2007 samples and 256 features.
- g50c: g50c is a binary dataset, and each class is generated by a 50-D multivariate Gaussian distribution [13].
- RNA-seq: It is a multi-class dataset about cancers, which has different types of tumors: BRCA, KIRC, COAD, LUAD, and PRAD. It has 801 samples and 20,531 features, and every attribute is RNA-Seq gene expression levels measured by the Illumina HiSeq platform.

To evaluate the performance of CSRGELM in practical applications, we apply CSRGELM to the cancer classification. In recent years, cancer has become the biggest threat to human health. The most effective way to treat cancers has always been to develop different treatments for different types of cancers. Therefore, the improvement of cancer classification is crucial to the progress of cancer treatments [32]. In this paper, four integrated TCGA datasets are used in the experiments. As known as the world's largest cancer genome database, the TCGA database has immeasurable values in the field of cancer research [33]. There are several types of cancer data included in the TCGA database. The details of benchmark datasets are listed in Table 2.

**Table 2:** Details of the benchmark datasets

Datasets	#Classes	#Samples	#Training	#Testing	#Features
Iris	3	150	120	30	4
COIL20	10	1440	1152	288	1024
USPST	20	2007	1605	402	256
g50c	2	550	440	110	50
RNA-seq	5	801	601	200	20,531

In the experiments, each integrated dataset is a combination of data from two or more cancers. In the integration process, to reduce the sample imbalance rate and ensure the credibility of the experimental results, we remove all normal samples and integrate only the disease samples of each cancer for classification experiments. Tables 3 and 4 list the information about the cancer data used in our experiments.

**Table 3:** The full name, abbreviation, and symbol for each cancer

Cancer name	Abbreviation	Symbol
Colon adenocarcinoma	COAD	C
Esophageal carcinoma	ESCA	E
Pancreatic adenocarcinoma	PAAD	P
Head and Neck squamous cell carcinoma	HNSC	H
Cholangiocarcinoma	CHOL	C_2_

**Table 4:** Information of the integrated datasets

Datasets	#Classes	#Samples	#Training	#Testing	#Features
CE	2	445	356	89	20,502
EHP	3	757	606	151	20,502
CEHP	4	1019	815	204	20,502
CEHPC_2_	5	1055	844	211	20,502

### Convergence and sensitivity

**Fig. 2:**
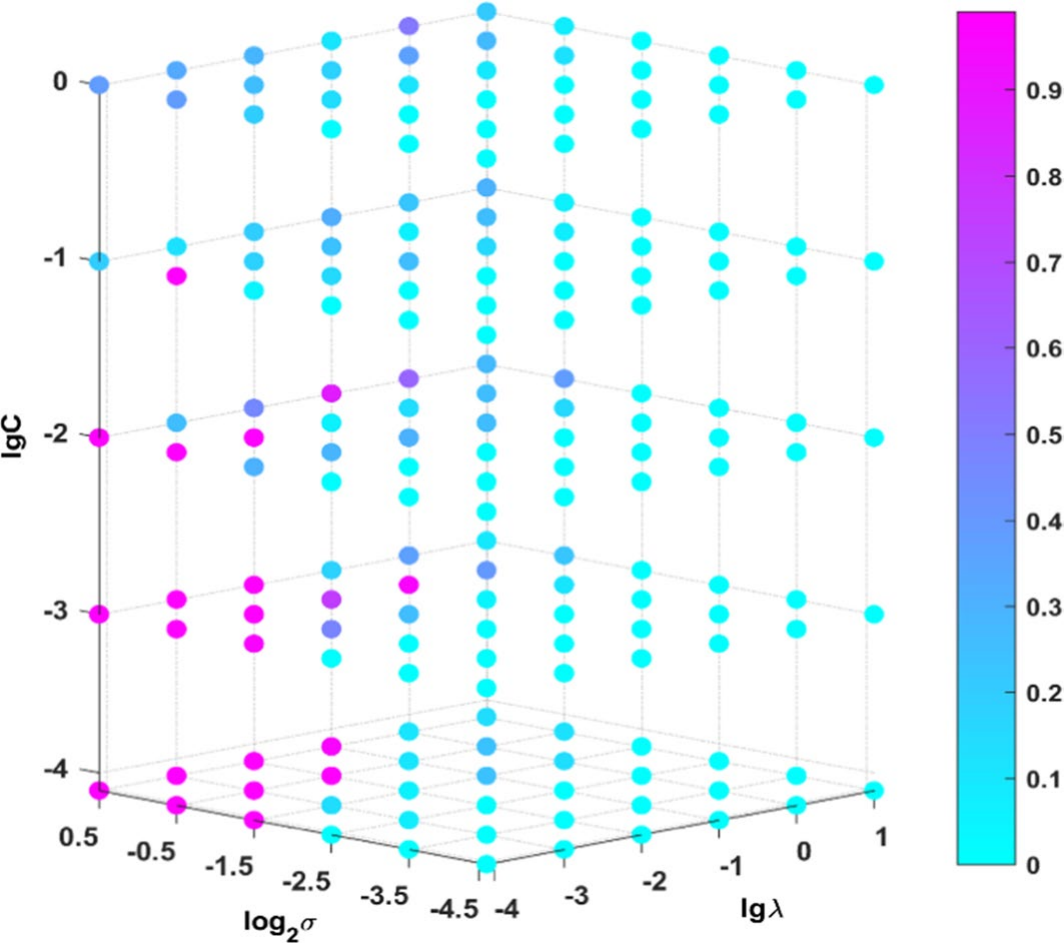
Parameter sensitivity of CSRGELM on COIL20

**Fig. 3:**
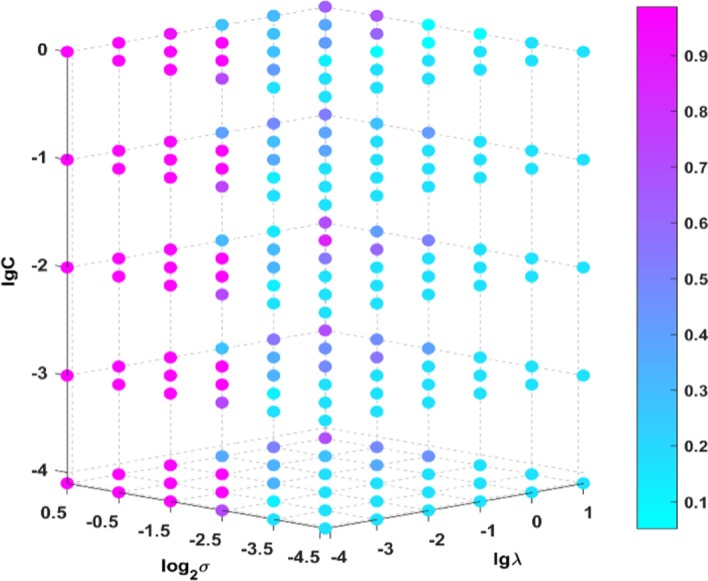
Parameter sensitivity of CSRGELM on CEHP

There are four parameters ($\sigma , \, \lambda , \, C, \, L$) that need to be turned in the experiments, and different combinations of parameters may produce different classification effects. Hence, ten fold cross-validation and grid search are used to find the optimal combination of parameters. Besides, the selection range of the parameter $\sigma$ is $\left( {2^{ - 4.5} , \ldots ,2^{4.5} } \right),$ $\lambda$ and $C$ are set as $\left( {10^{ - 4} ,10^{ - 3} , \ldots ,10^{5} } \right),$ and $L$ is set as $\left( {100,200, \ldots ,2000} \right)$. Taking datasets COIL20 and CEHP as examples, Figs. 2 and 3 depict the sensitivity of CSRGELM to different parameters. Because there are so many different combinations of parameters, we only show the first 180. As shown in the 4-D figures, the X-axis represents the range of $\lambda$, the Y-axis represents the range of $\sigma$, and the Z-axis represents the range of ${\text{C}}$. Each point in the figure represents the classification accuracy obtained by different parameter combinations. A conclusion can be drawn from Figs. 2 and 3 that CSRGELM is sensitive to $\sigma$ and ${\text{C,}}$ while it is insensitive to $\lambda .$ For the benchmark datasets, when $\sigma > 2^{ - 2.5}$ and $C < 10^{ - 1} ,$ the classification performance of CSRGELM is better. And for TCGA datasets, when $\sigma \ge 2^{ - 2.5}$ and $C \ge 10^{ - 4} ,$ the classification performance of CSRGELM is better.

**Fig. 4:**
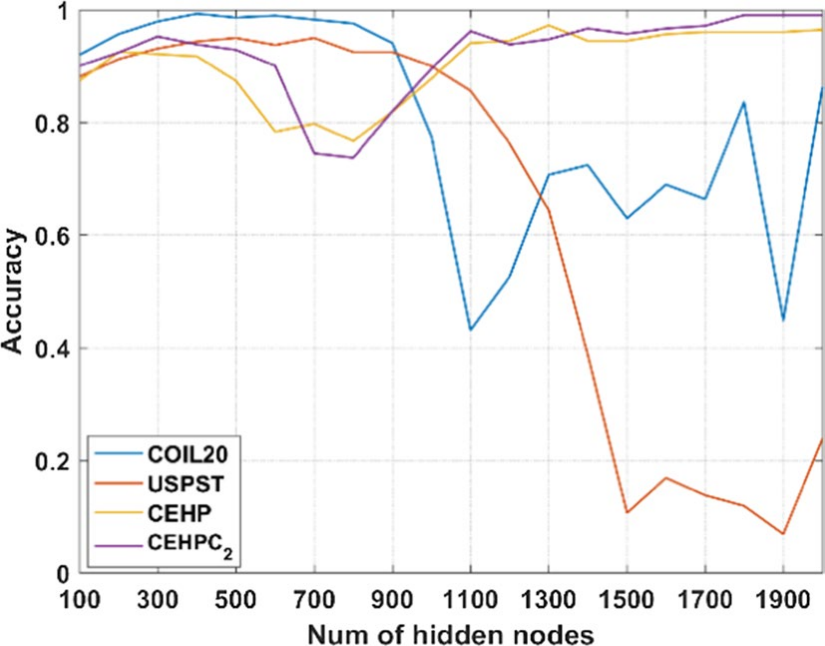
The influence of the number of hidden layer nodes on classification results

Taking four datasets as the examples, we also show the effect of the number of hidden layer nodes on classification performance in Fig. 4. It is obvious that with the increase of the number of hidden layer nodes, the classification performance of CSRGELM on the benchmark dataset fluctuates greatly. On the TCGA dataset, however, CSRGELM can obtain good classification results.

**Fig. 5:**
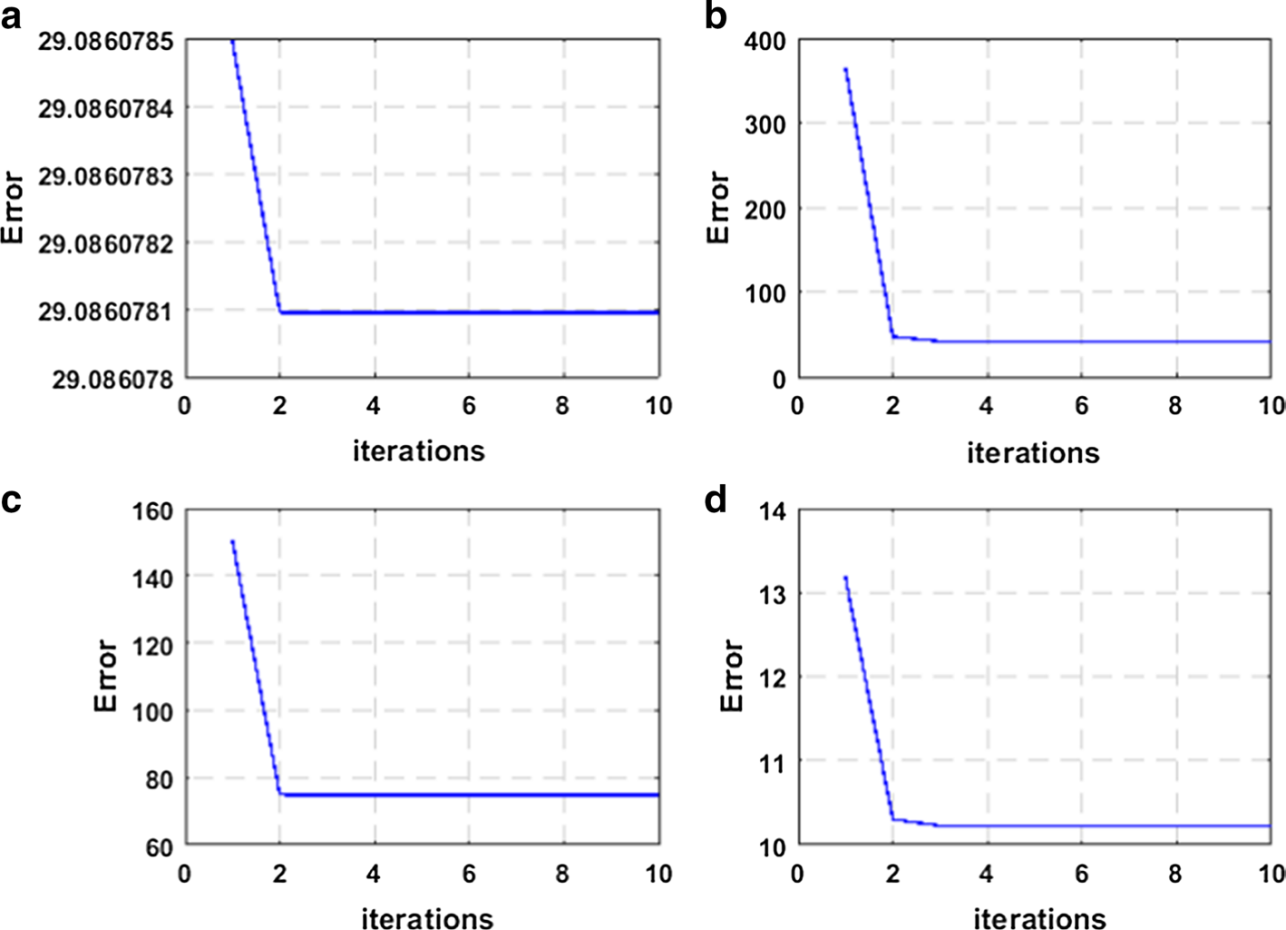
Convergence curve of CSRGELM on benchmark datasets **a** Iris, **b** COIL20, **c** USPST, **d** g50c

**Fig. 6:**
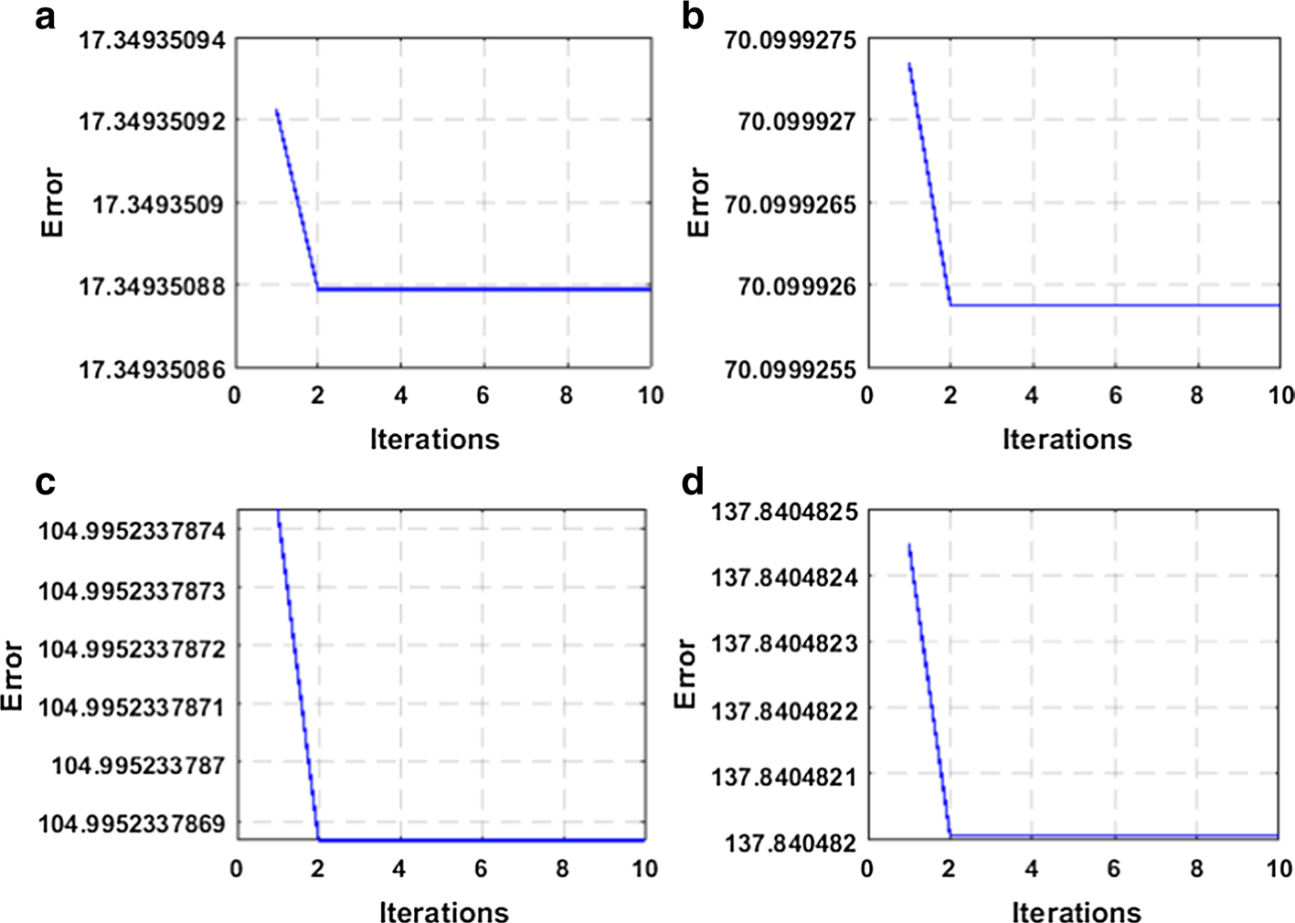
Convergence curve of CSRGELM on TCGA datasets **a** CE, **b** EHP, **c** CEHP, **d** CEHPC_2_

Besides, *L*_2,1_-norm and correntropy induced loss are introduced to our method, and their iterative optimization is more complicated. So, an iterative optimization algorithm is designed to solve the above optimization problem. As shown in Figs. 5 and 6, we plot the convergence curves to prove the convergence of the method. In the experiments, we assume that the method will converge after 40 iterations. And it's worth noting that CSRGELM can achieve convergence after 10 iterations. This can prove that the convergence rate of the method is relatively fast, and our iterative optimization algorithm is very efficient.

### Classification results on benchmark datasets and TCGA datasets

In this sub-section, the classification results of every method are provided. On every dataset, each method runs 20 times, and the average results and variance of the 20 classification results are listed in Tables 5 and 6. Besides, the running time of each method on different datasets is also listed in Tables 7 and 8. The best results are highlighted in italics.

**Table 5:** Classification results on benchmark datasets (± variance)

Datasets	Evaluations	Iris (*L* = 100)	COIL20 (*L* = 500)	USPST (*L* = 500)	g50c (*L* = 1000)	RNA-seq (*L* = 200)
RELM	Acc	0.9511 ± 0.0021	0.9763 ± 0.0001	0.9229 ± 0.0000	0.9001 ± 0.0004	0.9275 ± 0.0021
	Pre	0.9546 ± 0.0015	0.9753 ± 0.0004	0.9175 ± 0.0000	0.8937 ± 0.0014	0.9165 ± 0.0029
	Recall	0.9495 ± 0.0021	0.9752 ± 0.0005	0.9140 ± 0.0000	0.9202 ± 0.0033	0.9279 ± 0.0012
	F-mea	0.9459 ± 0.0025	0.9742 ± 0.0005	0.9150 ± 0.0000	*0.9146* ± *0.0002*	0.9205 ± 0.0020
*L*_21_-RFELM	Acc	0.9622 ± 0.0006	0.9815 ± 0.0000	0.9337 ± 0.0002	0.8210 ± 0.0010	0.9525 ± 0.0003
	Pre	0.9608 ± 0.0005	0.9823 ± 0.0000	0.9253 ± 0.0002	0.8407 ± 0.0034	0.9478 ± 0.0014
	Recall	0.9650 ± 0.0004	0.9812 ± 0.0000	0.9251 ± 0.0002	0.8008 ± 0.0018	0.9531 ± 0.0002
	F-mea	0.9604 ± 0.0005	0.9807 ± 0.0000	0.9240 ± 0.0002	0.8186 ± 0.0011	0.9497 ± 0.0002
LR21ELM	Acc	0.9556 ± 0.0035	0.9814 ± 0.0000	0.9401 ± 0.0000	0.8150 ± 0.0023	0.9425 ± 0.0036
	Pre	0.9594 ± 0.0019	0.9819 ± 0.0000	0.9341 ± 0.0001	0.8818 ± 0.0144	0.9445 ± 0.0029
	Recall	0.9587 ± 0.0025	0.9803 ± 0.0000	0.9325 ± 0.0000	0.8040 ± 0.0164	0.9332 ± 0.0065
	F-mea	0.9535 ± 0.0036	0.9799 ± 0.0000	0.9324 ± 0.0001	0.8423 ± 0.0057	0.9379 ± 0.0046
CELM	Acc	0.9634 ± 0.0002	0.9865 ± 0.0000	0.9306 ± 0.0001	0.8674 ± 0.0002	0.9400 ± 0.0002
	Pre	0.9702 ± 0.0002	0.9867 ± 0.0000	0.9215 ± 0.0001	0.8242 ± 0.0003	0.9458 ± 0.0001
	Recall	0.9641 ± 0.0001	0.9851 ± 0.0000	0.9227 ± 0.0001	*0.9344* ± *0.0002*	0.9358 ± 0.0001
	F-mea	0.9711 ± 0.0002	0.9853 ± 0.0000	0.9205 ± 0.0001	0.8758 ± 0.0002	0.9405 ± 0.0002
CSRGELM	Acc	*0.9788* ± *0.0000*	*0.9899* ± *0.0000*	*0.9513* ± *0.0005*	*0.9084* ± *0.0002*	*0.9625* ± *0.0002*
	Pre	*0.9745* ± *0.0000*	*0.9897* ± *0.0000*	*0.9346* ± *0.0001*	*0.8964* ± *0.0003*	*0.9626* ± *0.0001*
	Recall	*0.9747* ± *0.0000*	*0.9892* ± *0.0000*	*0.9325* ± *0.0001*	0.9152 ± 0.0005	*0.9631* ± *0.0002*
	F-mea	*0.9735* ± *0.0000*	*0.9891* ± *0.0000*	*0.9325* ± *0.0001*	0.9059 ± 0.0002	*0.9622* ± *0.0001*

**Table 6:** Classification results on TCGA datasets (± variance)

Datasets	Evaluations	CE (*L* = 1000)	EHP (*L* = 1000)	CEHP (*L* = 2000)	CEHPC_2_ (*L* = 2000)
RELM	Acc	0.9638 ± 0.0012	0.9589 ± 0.0003	0.9545 ± 0.0012	0.9623 ± 0.0010
	Pre	0.9714 ± 0.0003	0.9604 ± 0.0005	0.9434 ± 0.0010	0.9564 ± 0.0016
	Recall	0.9846 ± 0.0002	0.9508 ± 0.0003	0.9510 ± 0.0009	0.9584 ± 0.0015
	F-mea	0.9778 ± 0.0001	0.9546 ± 0.0004	0.9511 ± 0.0010	0.9564 ± 0.0018
*L*_21_-RFELM	Acc	0.9783 ± 0.0005	0.9571 ± 0.0001	0.9547 ± 0.0001	0.9753 ± 0.0001
	Pre	0.9714 ± 0.0012	0.9535 ± 0.0001	0.9668 ± 0.0005	0.9747 ± 0.0002
	Recall	0.9905 ± 0.0000	0.9426 ± 0.0004	0.9648 ± 0.0005	0.9751 ± 0.0002
	F-mea	0.9805 ± 0.0004	0.9478 ± 0.0003	0.9642 ± 0.0005	0.9745 ± 0.0002
LR21ELM	Acc	0.9823 ± 0.0004	0.9383 ± 0.0005	0.9403 ± 0.0002	0.9667 ± 0.0002
	Pre	0.9878 ± 0.0004	0.9393 ± 0.0005	0.9422 ± 0.0002	0.9718 ± 0.0000
	Recall	0.9877 ± 0.0004	0.9383 ± 0.0007	0.9292 ± 0.0004	0.9605 ± 0.0005
	F-mea	0.9865 ± 0.0003	0.9255 ± 0.0010	0.9273 ± 0.0004	0.9653 ± 0.0002
CELM	Acc	0.9062 ± 0.0046	0.9741 ± 0.0000	0.9646 ± 0.0000	0.9767 ± 0.0001
	Pre	0.9068 ± 0.0026	0.9691 ± 0.0000	*0.9722* ± *0.0000*	0.9706 ± 0.0000
	Recall	0.9454 ± 0.0042	0.9704 ± 0.0000	0.9690 ± 0.0000	*0.9857* ± *0.0001*
	F-mea	0.9306 ± 0.0027	*0.9796* ± *0.0000*	*0.9707* ± *0.0000*	0.9778 ± 0.0000
CSRGELM	Acc	*0.9964* ± *0.0001*	*0.9834* ± *0.0001*	*0.9709* ± *0.0000*	*0.9782* ± *0.0001*
	Pre	*0.9956* ± *0.0002*	*0.9860* ± *0.0000*	0.9678 ± 0.0000	*0.9813* ± *0.0001*
	Recall	*0.9970* ± *0.0002*	*0.9741* ± *0.0000*	*0.9695* ± *0.0000*	0.9774 ± 0.0003
	F-mea	*0.9963* ± *0.0001*	*0.9796* ± *0.0002*	0.9685 ± 0.0000	*0.9790* ± *0.0002*

**Table 7:** Training time of every method on benchmark datasets (± variance)

Datasets	RELM	*L*_21_-RFELM	LR21ELM	CELM	CSRGELM
Iris	*0.0020* ± *0.0000*	0.0197 ± 0.0004	0.0752 ± 0.0003	0.0221 ± 0.0001	0.0834 ± 0.0021
COIL20	*0.0366* ± *0.0000*	1.3369 ± 0.0001	2.9500 ± 0.0002	3.2786 ± 0.0599	6.2550 ± 0.0001
USPST	*0.0483* ± *0.0001*	0.4450 ± 0.0001	4.9219 ± 0.0242	5.1551 ± 0.0046	8.1832 ± 0.0010
g50c	*0.0055* ± *0.0001*	0.1535 ± 0.0001	0.4197 ± 0.0389	0.0962 ± 0.0038	0.2858 ± 0.1133
RNA-seq	*0.0070* ± *0.0000*	1.0653 ± 0.0001	1.9405 ± 0.4001	0.4083 ± 0.0003	1.4773 ± 0.0891

**Table 8:** Training time of every method on TCGA datasets (± variance)

Datasets	RELM	L21-RFELM	LR21ELM	CELM	CSRGELM
CE	*0.0108* ± *0.0000*	0.5854 ± 0.0004	0.8893 ± 0.0001	1.2014 ± 0.0001	3.4048 ± 0.0001
EHP	*0.0402* ± *0.0001*	1.2358 ± 0.0001	1.3432 ± 0.0003	3.1345 ± 0.0008	6.5448 ± 0.1786
CEHP	*0.0521* ± *0.0001*	4.0034 ± 0.3392	1.6710 ± 0.0005	8.4452 ± 0.6172	31.2409 ± 0.7403
CEHPC_2_	*0.0907* ± *0.0010*	4.6731 ± 0.0174	6.3159 ± 0.2753	9.1278 ± 0.0033	31.7088 ± 0.0013

A conclusion can be easily drawn that, both on the benchmark datasets and the integrated TCGA datasets, our method can get better results than other methods, or at least have competitive results. By evaluating each method using different evaluation measures, we can see that our method always gets a competitive result. Compared with RELM, *L*_2,1_-RFELM, LR21ELM, and CELM, CSRGELM obtains better results in most cases. In terms of running time, RELM can complete the training of the network model in the shortest time because there is no iterative adjustment. Compared with other methods, CSRGELM requires the most running time. According to the analysis, in addition to constantly iterating to optimize the output weight, the calculation of ${\mathbf{H}}^{T} {\mathbf{ZH}}$ or ${\mathbf{ZHH}}^{T}$ also takes a lot of time. How to shorten the training time is also a problem we need to study in the future.

**Fig. 7:**
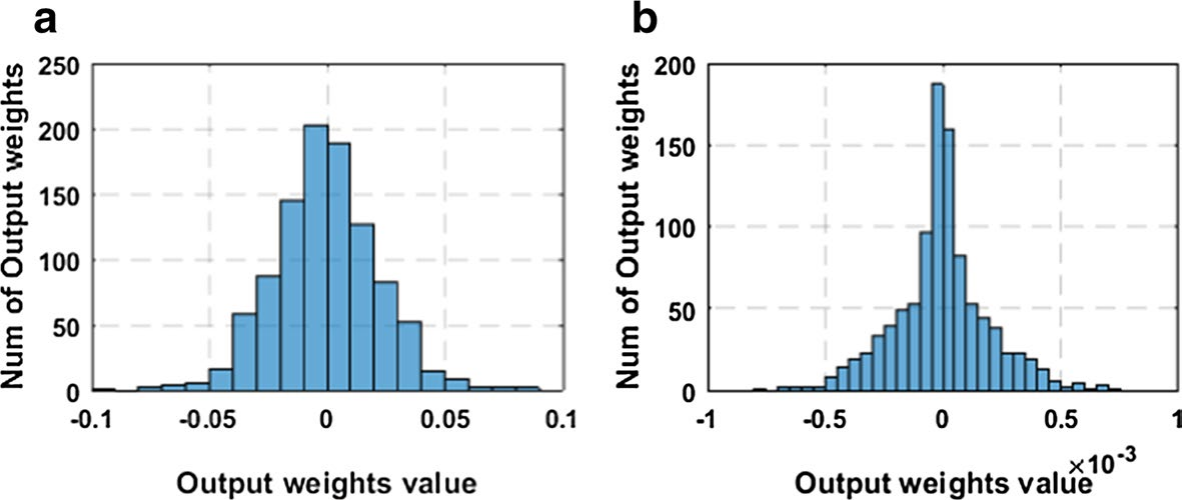
Output weight distribution on CE **a** CELM, **b** CSRGELM

**Fig. 8:**
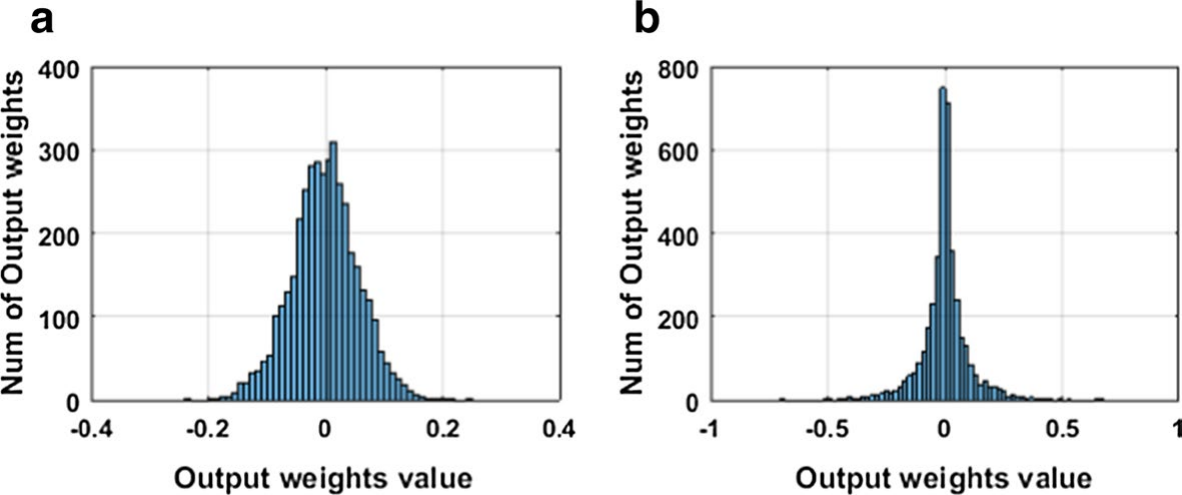
Output weight distribution on CEHP **a** CELM, **b** CSRGELM

As stated in the previous section, *L*_2,1_-norm is applied to the output weight matrix as a sparse regularization constraint. To prove the validity of the sparse constraint and the sparseness of the output weight matrix, we analyze the weight distribution of CSRGELM and CELM. Figures 7 and 8 show the output weight distribution of CELM and CSRGELM on CE and CEHP.

From Figs. 7 and 8, we can conclude that the distribution of the elements of the output weight matrix is almost concentrated around zero. This proves that by the constraint of *L*_2,1_-norm to ${{\varvec{\upbeta}}}$, we can obtain a sparser network model, which makes the model easier to explain and saves storage space and resources. In the neural network model, a sparse network model can achieve feature selection, and then we can remove the unrelated hidden layer nodes to get a more simplified and efficient neural network model.

## Discussion

Our method is applied to the sample classification problems, and the generalization performance is better than other methods. The main reason is that the non-convex function of the correntropy-induced loss is introduced to improve the robustness. CSRGELM is more efficient and accurate than CELM because of the introduction of the graph regularization. What’s more, the *L*_2,1_-norm regularization constraint has also contributed to the improvement of classification performance. Although in another method LR21ELM [9], the *L*_2,1_-norm is also used as a loss function to improve the robustness, from the experimental results, in most cases, the robustness of the *L*_2,1_-norm is weaker than the correntropy induced loss. In other words, correntropy induced loss based methods can effectively reduce the negative influence of noise and outliers on classification results. At the same time, the introduction of the graph regularization can preserve the local structural information of data. The effective combination of them can not only improve the classification performance, but also improve the generalization ability of the model.

The introduction of *L*_2,1_-norm regularization tends to produce a structural sparsity. It is capable of reducing some rows of the output weight matrix to zero and simplify the inherent complexity of the neural network model. The results of Figs. 7 and 8 also prove the validity of the *L*_2,1_-norm regularization.

## Conclusions

In this paper, we propose a new method named correntropy induced loss based sparse robust graph regularized extreme learning machine (CSRGELM) and apply it to the classification problems of cancer samples. The introduction of correntropy induced loss weakens the influence of noise and outliers on the classification performance and improves the robustness of the method. As a powerful sparse regularization constraint, *L*_2,1_-norm is used to constrain the output weight matrix, which can reduce the complexity of the network model. Besides, the graph regularization is introduced to preserve the local manifold structure between data and reduce the loss of information. To solve the above optimization problem, we propose an efficient iterative optimization algorithm, and the computational complexity of the algorithm is also proved. Whether on the benchmark datasets or the TCGA integrated datasets, the classification performance and generalization performance of CSRGELM are comparable to other methods. In future work, we will still conduct in-depth research on the robustness of ELM and apply it to the field of bioinformatics.

## Methods

### RELM

Huang et al. proposed the regularized-extreme learning machine (RELM) in [5] and proved its good performance in classification or regression problems. For a dataset $\left\{ {{\mathbf{X}},{\mathbf{T}}} \right\} = \left\{ {{\mathbf{x}}_{i} ,{\mathbf{t}}_{i} } \right\}_{i = 1}^{N} \in {\mathbb{R}}^{N \times m} ,$ where $N$ is the number of samples and $m$ is the number of features. The objective function of RELM can be expressed as:

5 $$\mathop {\min }\limits_{{{{\varvec{\upbeta}}}, \, {{\varvec{\upxi}}}}} \frac{1}{2}\left\| {{\varvec{\upbeta}}} \right\|^{2} + \frac{\gamma }{2}\sum\nolimits_{i = 1}^{N} {\left\| {{{\varvec{\upxi}}}_{i} } \right\|^{2} } ,\,{\text{s.t.}}\,{{\varvec{\upxi}}}_{i}^{T} = {\mathbf{t}}_{i}^{T} - {\mathbf{h}}\left( {{\mathbf{x}}_{i} } \right){{\varvec{\upbeta}}},\,i = 1, \ldots , \, N,$$

where $\gamma$ is a regularization parameter, and ${{\varvec{\upxi}}}_{i}$ is the error vector of $i$-th sample. ${\mathbf{T}}$ is the target label matrix. Substituting constraints into Eq. (5), we get the following unconstrained optimization problem:

6 $$\mathop {\min }\limits_{{{\varvec{\upbeta}}}} \frac{1}{2}\left\| {{\varvec{\upbeta}}} \right\|^{2} + \frac{\gamma }{2}\left\| {{\mathbf{T}} - {\mathbf{H\beta }}} \right\|^{2} {.}$$

Let $L$ be the number of hidden nodes, if $N \ge L,$ the solution of ${{\varvec{\upbeta}}}$ can be obtained by calculating the partial derivative of Eq. (6) and setting it to zero:

7 $${{\varvec{\upbeta}}} - \gamma {\mathbf{H}}^{T} \left( {{\mathbf{T}} - {\mathbf{H\beta }}} \right) = 0,$$

and

8 $${{\varvec{\upbeta}}} = \left( {\gamma {\mathbf{H}}^{T} {\mathbf{H}} + {\mathbf{I}}_{L} } \right)^{ - 1} \gamma {\mathbf{H}}^{T} {\mathbf{T}},$$

where ${\mathbf{I}}_{L}$ is an identity matrix with dimension $L.$ If $N < L,$ ${{\varvec{\upbeta}}}$ can be calculated as:

9 $${{\varvec{\upbeta}}} = {\mathbf{H}}^{T} \left( {\gamma {\mathbf{HH}}^{T} + {\mathbf{I}}_{N} } \right)^{ - 1} \gamma {\mathbf{T}},$$

where ${\mathbf{I}}_{N}$ is an identity matrix with dimension $N.$ Finally, we get the solution of ${{\varvec{\upbeta}}}$:

10 $$\left\{ {\begin{array}{*{20}l} {{{\varvec{\upbeta}}} = \left( {\gamma {\mathbf{H}}^{T} {\mathbf{H}} + {\mathbf{I}}_{L} } \right)^{ - 1} \gamma {\mathbf{H}}^{T} {\mathbf{T}},} \hfill & {N \ge L.} \hfill \\ {{{\varvec{\upbeta}}} = {\mathbf{H}}^{T} \left( {\gamma {\mathbf{HH}}^{T} + {\mathbf{I}}_{N} } \right)^{ - 1} \gamma {\mathbf{T}},} \hfill & {N < L.} \hfill \\ \end{array} } \right.$$

### L_2,1_-RFELM

As a regularization constraint, Zhou et al. introduced the *L*_2,1_-norm to constrain the output weight matrix ${{\varvec{\upbeta}}}$ [34]. *L*_2,1_-norm regularization can generate row-sparsity, which can eliminate the redundant nodes and achieve the feature selection [35–37]. The mathematical model of *L*_2,1_-RFELM is:

11 $$\mathop {\min }\limits_{{{{\varvec{\upbeta}}}, \, {{\varvec{\upxi}}}}} \frac{1}{2}\left\| {{\varvec{\upbeta}}} \right\|_{2,1} + \frac{C}{2}\sum\nolimits_{i = 1}^{N} {\left\| {{{\varvec{\upxi}}}_{i} } \right\|^{2} } ,\,{\text{s.t.}}\,{{\varvec{\upxi}}}_{i}^{T} = {\mathbf{t}}_{i}^{T} - {\mathbf{h}}\left( {{\mathbf{x}}_{i} } \right){{\varvec{\upbeta}}},\,i = 1, \ldots ,N,$$

where $C$ is a parameter of the regularization term. Then, Eq. (11) can be rewritten as:

12 $$\ell = \frac{1}{2}{\text{Tr}} \left( {{{\varvec{\upbeta}}}^{T} {\mathbf{D\upbeta}}} \right) + \frac{C}{2}\left\| {{\mathbf{T}} - {\mathbf{H\upbeta }}} \right\|^{2} ,$$

where ${\mathbf{D}}$ is a diagonal matrix with $d_{ii} = {1 \mathord{\left/ {\vphantom {1 {\left( {2\left\| {{{\varvec{\upbeta}}}_{i} } \right\|_{2} } \right)}}} \right. \kern-\nulldelimiterspace} {\left( {2\left\| {{{\varvec{\upbeta}}}_{i} } \right\|_{2} } \right)}}.$ By computing the derivative of ${{\varvec{\upbeta}}}$ and setting it equal to zero, we have:

13 $${\mathbf{D\upbeta }} - C{\mathbf{H}}^{T} \left( {{\mathbf{T}} - {\mathbf{H\upbeta }}} \right) = 0.$$

According to the relationship between the number of samples and hidden layer nodes, there are two analytic solutions for ${{\varvec{\upbeta}}}$:

14 $$\left\{ {\begin{array}{*{20}l} {{{\varvec{\upbeta}}} = \left( {{\mathbf{D}} + C{\mathbf{H}}^{T} {\mathbf{H}}} \right)^{{{ - }1}} C{\mathbf{H}}^{T} {\mathbf{T}},} \hfill & {N \ge L,} \hfill \\ {{{\varvec{\upbeta}}} = C{\mathbf{D}}^{ - 1} {\mathbf{H}}^{T} \left( {{\mathbf{I}} + C{\mathbf{HD}}^{ - 1} {\mathbf{H}}^{T} } \right){\mathbf{T}},} \hfill & {N < L{.}} \hfill \\ \end{array} } \right.$$

### LR21ELM

In [9], Li et al. introduced the *L*_2,1_-norm to constrain both the error matrix ${{\varvec{\upxi}}}$ and the output weight matrix ${{\varvec{\upbeta}}}$, and proposed a robust sparse ELM method named LR21ELM. The objective function of LR21ELM is:

15 $$\mathop {\min }\limits_{{{{\varvec{\upbeta}}}, \, {{\varvec{\upxi}}}}} \left\| {{\varvec{\upbeta}}} \right\|_{2,1} + C\left\| {{\varvec{\upxi}}} \right\|_{2,1} ,\,{\text{s.t.}}\,{{\varvec{\upxi}}}_{i}^{T} = {\mathbf{t}}_{i}^{T} - {\mathbf{h}}\left( {{\mathbf{x}}_{i} } \right){{\varvec{\upbeta}}},\,i = 1, \ldots ,N{.}$$

Following the KKT theorem, the Lagrangian function of Eq. (15) is defined as:

16 $$\ell_{LR21ELM} = C\left\| {{\varvec{\upxi}}} \right\|_{2,1} + \left\| {{\varvec{\upbeta}}} \right\|_{2,1} - \sum\nolimits_{i = 1}^{N} {\sum\nolimits_{j = 1}^{m} {{{\varvec{\uptheta}}}_{ij} } } \left( {{\mathbf{h}}\left( {{\mathbf{x}}_{i} } \right){{\varvec{\upbeta}}} - {\mathbf{t}}_{ij} + {{\varvec{\upxi}}}_{ij} } \right),$$

where ${{\varvec{\uptheta}}}_{ij}$ is the Lagrange multiplier. Based on the solution in [38], Eq. (16) is equivalent to:

17 $$\ell_{LR21ELM} = C{\text{Tr}} \left( {{{\varvec{\upxi}}}^{T} {\mathbf{D}}_{1} {{\varvec{\upxi}}}} \right) + {\text{Tr}} \left( {{{\varvec{\upbeta}}}^{T} {\mathbf{D\upbeta }}} \right) - \sum\nolimits_{i = 1}^{N} {\sum\nolimits_{j = 1}^{m} {{{\varvec{\uptheta}}}_{ij} } } \left( {{\mathbf{h}}\left( {{\mathbf{x}}_{i} } \right){{\varvec{\upbeta}}} - {\mathbf{t}}_{ij} + {{\varvec{\upxi}}}_{ij} } \right),$$

where ${\mathbf{D}}_{1} = {1 \mathord{\left/ {\vphantom {1 {\left( {2\left\| {{{\varvec{\upxi}}}_{i} } \right\|_{2} } \right)}}} \right. \kern-\nulldelimiterspace} {\left( {2\left\| {{{\varvec{\upxi}}}_{i} } \right\|_{2} } \right)}},$ and ${\mathbf{D}} = {1 \mathord{\left/ {\vphantom {1 {\left( {2\left\| {{{\varvec{\upbeta}}}_{i} } \right\|_{2} } \right)}}} \right. \kern-\nulldelimiterspace} {\left( {2\left\| {{{\varvec{\upbeta}}}_{i} } \right\|_{2} } \right)}}.$ According to Eq. (17), the optimal conditions can be written as:

18 $$\frac{{\partial \ell_{LR21ELM} }}{{\partial {{\varvec{\uptheta}}}_{i} }} = 0 \Rightarrow {\mathbf{H\upbeta }} - {\mathbf{T}} + {{\varvec{\upxi}}} = 0,$$

19 $$\frac{{\partial \ell_{LR21ELM} }}{{\partial {{\varvec{\upbeta}}}_{i} }} = 0 \Rightarrow {\mathbf{D\upbeta }} = {\mathbf{H}}^{T} {{\varvec{\uptheta}}},$$

20 $$\frac{{\partial \ell_{LR21ELM} }}{{\partial {{\varvec{\upxi}}}_{i} }} = 0 \Rightarrow {{\varvec{\uptheta}}} = C{\mathbf{D}}_{1} {{\varvec{\upxi}}}.$$

If $N < L,$ by substituting Eq. (19) and Eq. (20) into Eq. (18), we have:

21 $${{\varvec{\uptheta}}} = \left( {{\mathbf{HD}}^{ - 1} {\mathbf{H}}^{T} + \frac{{{\mathbf{D}}_{1}^{ - 1} }}{C}} \right)^{ - 1} {\mathbf{T}}.$$

According to Eq. (19), we have:

22 $${{\varvec{\upbeta}}} = {\mathbf{D}}^{ - 1} {\mathbf{H}}^{T} \left( {{\mathbf{HD}}^{ - 1} {\mathbf{H}}^{T} + \frac{{{\mathbf{D}}_{1}^{ - 1} }}{C}} \right)^{ - 1} {\mathbf{T}}.$$

And if $N \ge L,$ by combining Eq. (19) with Eq. (20), we have:

23 $${{\varvec{\upxi}}} = \frac{{\left( {{\mathbf{H}}^{T} {\mathbf{D}}_{1} } \right)^{\dag } {\mathbf{D\upbeta }}}}{C}.$$

Substituting Eq. (23) into Eq. (18), we obtain an alternative solution of ${{\varvec{\upbeta}}}$:

24 $${{\varvec{\upbeta}}} = \left( {{\mathbf{H}}^{T} {\mathbf{D}}_{1} {\mathbf{H}} + \frac{{\mathbf{D}}}{C}} \right)^{ - 1} {\mathbf{H}}^{T} {\mathbf{D}}_{1} {\mathbf{T}}.$$

So, the analytic solution of ${{\varvec{\upbeta}}}$ is:

25 $$\left\{ {\begin{array}{*{20}l} {{{\varvec{\upbeta}}} = \left( {{\mathbf{H}}^{T} {\mathbf{D}}_{1} {\mathbf{H}} + \frac{{\mathbf{D}}}{C}} \right)^{ - 1} {\mathbf{H}}^{T} {\mathbf{D}}_{1} {\mathbf{T}}.} \hfill & {N \ge L,} \hfill \\ {{{\varvec{\upbeta}}} = {\mathbf{D}}^{ - 1} {\mathbf{H}}^{T} \left( {{\mathbf{HD}}^{ - 1} {\mathbf{H}}^{T} + \frac{{{\mathbf{D}}_{1}^{ - 1} }}{C}} \right)^{ - 1} {\mathbf{T}}.} \hfill & {N < L{.}} \hfill \\ \end{array} } \right.$$

### Graph regularization

Graph regularization framework [39] has been widely used in semi-supervised learning [13] and unsupervised learning [40–43]. In the process of data processing, the graph regularization can preserve the local manifold structure between data, so that the structural information can be extracted, which is beneficial to clustering or classification problems. In mathematics, the expression of graph regularization is as follows:

26 $${\mathbf{Q}}_{gL} = \frac{1}{2}\sum\nolimits_{i,j} {{\mathbf{W}}_{i,j} \left\| {{\text{P}} \left( {{\mathbf{t}}|{\mathbf{x}}_{i} } \right) - {\text{P}} \left( {{\mathbf{t}}|{\mathbf{x}}_{j} } \right)} \right\|}^{2} ,$$

where ${\text{P}} \left( {{\mathbf{t}}|{\mathbf{x}}_{i} } \right)$ and ${\text{P}} \left( {{\mathbf{t}}|{\mathbf{x}}_{j} } \right)$ are conditional probabilities, and ${\mathbf{W}} = \left[ {{\mathbf{W}}_{i,j} } \right]$ is the similarity matrix. Equation (26) is equal to

27 $${\mathbf{Q}}_{gL}^{^{\prime}} = \frac{1}{2}\sum\nolimits_{i,j} {{\mathbf{W}}_{i,j} \left\| {{\mathbf{t}}_{i} - {\mathbf{t}}_{j} } \right\|}^{2} ,$$

where ${\mathbf{t}}_{i}$ and ${\mathbf{t}}_{j}$ are predictions of ${\mathbf{x}}_{i}$ and ${\mathbf{x}}_{j}$, respectively. And the matrix form of Eq. (27) is:

28 $${\mathbf{Q}}_{gL}^{^{\prime}} = {\text{Tr}} \left( {{\mathbf{T}}^{T} {\mathbf{ZT}}} \right),$$

where ${\mathbf{T}}$ is the prediction matrix, ${\text{Tr}} ( \bullet)$ is the trace norm and ${\mathbf{Z}} = {\mathbf{D}} - {\mathbf{W}}$ is the graph Laplacian matrix. ${\mathbf{D}}$ is a diagonal matrix with $d_{ii} = \sum\nolimits_{j} {{\mathbf{W}}_{i,j} .}$

### Proposed CSRGELM

In practical applications, the dataset usually includes a lot of noise and outliers, which will cause serious interference to the experiment results, so as to obtain inaccurate results [44]. Due to the noise and outliers, the classification effect of ELM always fails to meet the expectation. A large number of conclusions have proved that the introduction of the graph regularization in ELM method can effectively improve the classification performance or feature extraction ability of the algorithm [45, 46]. Therefore, it is necessary to develop a robust and efficient method for outliers and noise.

In this section, we propose a novel method which is named correntropy induced loss based sparse robust graph regularized extreme learning machine (CSRGELM). The correntropy induced loss function is introduced to replace the square loss, which can effectively improve the robustness of the method. And in our method, the *L*_2,1_-norm is used to constrain the output weight matrix ${{\varvec{\upbeta}}}$. As an adaptive sparse regularization term, *L*_2,1_-norm is used to constrain the output weight matrix, which can generate row sparsity, eliminate redundant hidden layer nodes and simplify the structure of the neural network. In recent years, how to use local consistency of data for learning to improve the performance of machine learning methods that has attracted researchers' attention [45]. Based on the theory that similar samples should have similar properties, the graph regularization is combined with our method to preserve the local structural information, which may improve the classification performance of the method [13, 47]. We use the label information of the training sample to construct an adjacent graph, and the regularization term of the graph is integrated to constrain the output weight matrix, so as to learn the similar output of similar samples.

#### The objective function of CSRGELM

This section introduces the objective function of CSRGELM. For a dataset $\left\{ {{\mathbf{X}}_{train} , \, {\mathbf{T}}_{train} } \right\} = \left\{ {{\mathbf{x}}_{i} , \, {\mathbf{t}}_{i} } \right\}_{i = 1}^{N} \in {\mathbb{R}}^{N \times m} ,$ ${\mathbf{T}}_{train}$ is the label matrix of ${\mathbf{X}}_{train}$, $N$ is the number of samples, and $m$ is the number of features. The mathematical model of CSRGELM can be expressed as:

29 $$\begin{aligned} & {\text{F}} \left( {{\varvec{\upbeta}}} \right) = \mathop {\min }\limits_{{{\varvec{\upbeta}}}} \sum\nolimits_{i = 1}^{N} {\left( {1 - \exp \left( { - \frac{{{{\varvec{\upxi}}}_{i}^{2} }}{{2\sigma^{2} }}} \right)} \right)} + \frac{\lambda }{2}\left\| {{\varvec{\upbeta}}} \right\|_{2,1} + \frac{C}{2}{\text{Tr}} \left( {\left( {{\mathbf{H\upbeta }}} \right)^{T} {\mathbf{ZH\upbeta }}} \right), \\ & \quad s.t.\quad {\mathbf{h}}\left( {{\mathbf{x}}_{i} } \right){{\varvec{\upbeta}}} = {\mathbf{t}}_{i}^{T} - {{\varvec{\upxi}}}_{i}^{T} ,i = 1, \ldots ,N. \\ \end{aligned}$$

In Eq. (29), ${{\varvec{\upxi}}}_{i}$ is the error vector, $\sigma$ is the bandwidth and ${\mathbf{Z}}$ is the graph Laplacian matrix. $\lambda$ and $C$ are regularization parameters, respectively. Since Eq. (29) is not a convex function, it can’t be solved by a commonly used optimization method. According to the solution process in [23], we can effectively solve the optimization problem of non-convex functions.

#### The optimization of CSRGELM

Since the correntropy induced loss is a differentiable and smooth function, the gradient optimization algorithm can be employed [23]. However, the gradient-based optimization algorithm converges slowly, so we use the half-quadratic optimization algorithm to solve the optimization problem of CSRGELM.

Firstly, we should define a convex function as:

30 $${\text{f}} \left( \tau \right) = - \tau \log \left( \tau \right) + \tau ,$$

where $\tau < 0.$ Following the definition and solution of conjugate function in [48]: If we define a differentiable function: $\psi \left( x \right): \, {\mathbb{R}}^{n} \to {\mathbb{R}},$ the conjugate function $\psi^{*} \left( x \right): \, {\mathbb{R}}^{n} \to {\mathbb{R}}$ can be expressed as: $\psi^{*} \left( x \right) = \mathop {\sup }\limits_{p} \left( {px - \psi \left( p \right)} \right).$ And if $\psi \left( x \right)$ is a convex function, we can obtain that $\left( {\psi^{*} \left( x \right)} \right)^{*} = \psi \left( x \right)$ [49]. we can obtain the conjugate function of Eq. (30):

31 $${\text{f}}^{*} \left( \upsilon \right) = \sup {\text{f}}^{{\prime }} \left( \tau \right),$$

and

32 $${\text{f}}^{{\prime }} \left( \tau \right) = \upsilon \tau - {\text{f}} \left( \tau \right) = \upsilon \tau + \tau \log \left( { - \tau } \right) - \tau .$$

By letting ${{d{\text{f}}^{{\prime }} \left( \tau \right)} \mathord{\left/ {\vphantom {{d{\text{f}}^{{\prime }} \left( \tau \right)} {d\tau }}} \right. \kern-\nulldelimiterspace} {d\tau }} = 0,$ the solution of Eq. (32) can be obtained:

33 $$\upsilon + \log \left( { - \tau } \right) = 0 \Rightarrow \tau = - \exp \left( { - \upsilon } \right) < 0.$$

Substituting Eq. (33) into Eq. (31), so Eq. (34) can be expressed as:

34 $${\text{f}}^{*} \left( \upsilon \right) = \exp \left( { - \upsilon } \right).$$

When we assume ${{\varvec{\upupsilon}}} = {{{{\varvec{\upxi}}}_{i}^{2} } \mathord{\left/ {\vphantom {{{{\varvec{\upxi}}}_{i}^{2} } {2\sigma^{2} }}} \right. \kern-\nulldelimiterspace} {2\sigma^{2} }},$ we will have

35 $${\text{f}}^{*} \left( {\frac{{{{\varvec{\upxi}}}_{i}^{2} }}{{2\sigma^{2} }}} \right) = \sup \left( {\frac{{{{\varvec{\upxi}}}_{i}^{2} }}{{2\sigma^{2} }}\tau + \tau \log \left( { - \tau } \right) - \tau } \right) = \exp \left( { - \frac{{{{\varvec{\upxi}}}_{i}^{2} }}{{2\sigma^{2} }}} \right).$$

As described in [23], the supremum is reached when $\tau = - \exp \left( { - \left( {{{{{\varvec{\upxi}}}_{i}^{2} } \mathord{\left/ {\vphantom {{{{\varvec{\upxi}}}_{i}^{2} } {2\sigma^{2} }}} \right. \kern-\nulldelimiterspace} {2\sigma^{2} }}} \right)} \right) < 0.$

Combining Eq. (35) with Eq. (29), and we can get hold of the following mathematical model:

36 $$\begin{gathered} {\text{F}}^{^{\prime}} \left( {{\varvec{\upbeta}}} \right) = \mathop {\min }\limits_{{{{\varvec{\upbeta}}},{{\varvec{\uptau}}}}} \sum\nolimits_{i = 1}^{N} {\left( {1 - \sup \left( {\frac{{{{\varvec{\upxi}}}_{i}^{2} }}{{2\sigma^{2} }}{{\varvec{\uptau}}}_{i} - {\text{f}} \left( {{{\varvec{\uptau}}}_{i} } \right)} \right)} \right)} + \frac{\lambda }{2}\left\| {{\varvec{\upbeta}}} \right\|_{2,1} + \frac{C}{2}{\text{Tr}} \left( {\left( {{\mathbf{H\upbeta }}} \right)^{T} {\mathbf{ZH\upbeta }}} \right), \hfill \\ s.t. \, {\mathbf{h}}\left( {{\mathbf{x}}_{i} } \right){{\varvec{\upbeta}}} = {\mathbf{t}}_{i}^{T} - {{\varvec{\upxi}}}_{i}^{T} , \, i = 1, \, \ldots , \, N, \hfill \\ \end{gathered}$$

where ${{\varvec{\uptau}}} = [{{\varvec{\uptau}}}_{1} , \, {{\varvec{\uptau}}}_{2} , \ldots {,}{{\varvec{\uptau}}}_{N} ]^{T} .$ Equation (36) can be rewritten as:

37 $$\begin{gathered} {\text{F}}^{^{\prime\prime}} \left( {{\varvec{\upbeta}}} \right) = \mathop {\min }\limits_{{{{\varvec{\upbeta}}},{{\varvec{\uptau}}}}} \left( {\sup \sum\nolimits_{i = 1}^{N} {\left( { - \frac{{{{\varvec{\upxi}}}_{i}^{2} }}{{2\sigma^{2} }}{{\varvec{\uptau}}}_{i} + {\text{f}} \left( {{{\varvec{\uptau}}}_{i} } \right)} \right) + \frac{\lambda }{2}\left\| {{\varvec{\upbeta}}} \right\|_{2,1} + \frac{C}{2}{\text{Tr}} \left( {\left( {{\mathbf{H\upbeta }}} \right)^{T} {\mathbf{ZH\upbeta }}} \right)} } \right), \hfill \\ s.t. \, {\mathbf{h}}\left( {{\mathbf{x}}_{i} } \right){{\varvec{\upbeta}}} = {\mathbf{t}}_{i}^{T} - {{\varvec{\upxi}}}_{i}^{T} , \, i = 1, \, \ldots , \, N. \hfill \\ \end{gathered}$$

Obviously, there are two variables that need to be optimized: ${{\varvec{\uptau}}}$ and ${{\varvec{\upbeta}}}$. Here we use a method of fixing one to optimize the other to solve Eq. (37).

1. *Fixed *${{\varvec{\upbeta}}}^{n}$* to optimize *${{\varvec{\uptau}}}^{n + 1} .$

For a given ${{\varvec{\upbeta}}}^{n} ,$ Eq. (37) can be expressed as:

38 $$\mathop {\min }\limits_{{{{\varvec{\uptau}}}^{n + 1} }} \sum\nolimits_{i = 1}^{N} {\left( { - \frac{{{{\varvec{\upxi}}}_{i}^{2} }}{{2\sigma^{2} }}{{\varvec{\uptau}}}_{i}^{n + 1} + {\text{f}} \left( {{{\varvec{\uptau}}}_{i}^{n + 1} } \right)} \right)} ,\,s.t.\,{\mathbf{h}}\left( {{\mathbf{x}}_{i} } \right){{\varvec{\upbeta}}}^{n} = {\mathbf{t}}_{i}^{T} - {{\varvec{\upxi}}}_{i}^{T} ,\,i = 1, \ldots ,N{.}$$

Substituting constraints into Eq. (38), we can get:

39 $$\mathop {\min }\limits_{{{{\varvec{\uptau}}}^{n + 1} }} \sum\nolimits_{i = 1}^{N} {\left( { - \frac{{\left( {{\mathbf{t}}_{i}^{T} - {\mathbf{h}}\left( {{\mathbf{x}}_{i} } \right){{\varvec{\upbeta}}}^{n} } \right)^{2} }}{{2\sigma^{2} }}{{\varvec{\uptau}}}_{i}^{n + 1} + {\text{f}} \left( {{{\varvec{\uptau}}}_{i}^{n + 1} } \right)} \right)} .$$

According to Eq. (32), the solution of Eq. (39) is:

40 $${{\varvec{\uptau}}}_{i}^{n + 1} = - \exp \left( { - \frac{{\left( {{\mathbf{t}}_{i}^{T} - {\mathbf{h}}\left( {{\mathbf{x}}_{i} } \right){{\varvec{\upbeta}}}^{n} } \right)^{2} }}{{2\sigma^{2} }}} \right),\,i = 1, \ldots ,N,$$

where $${{\varvec{\uptau}}}_{i}^{n + 1} < 0.$$

2 *Fixed *${{\varvec{\uptau}}}^{n + 1}$* to optimize *${{\varvec{\upbeta}}}^{n + 1} .$

For a given ${{\varvec{\uptau}}}^{n + 1} ,$ we focus on solving the problem as:

41 $$\begin{aligned} & \mathop {\min }\limits_{{{{\varvec{\upbeta}}}^{n + 1} }} \left( {\sum\nolimits_{i = 1}^{N} {\left( { - \frac{{{{\varvec{\uptau}}}_{i}^{n + 1} }}{{2\sigma^{2} }}{{\varvec{\upxi}}}_{i}^{2} } \right) + \frac{\lambda }{2}\left\| {{{\varvec{\upbeta}}}^{n + 1} } \right\|_{2,1} + \frac{C}{2}{\text{Tr}} \left( {\left( {{\mathbf{H\upbeta }}^{n + 1} } \right)^{T} {\mathbf{ZH\upbeta }}^{n + 1} } \right)} } \right), \\ & \quad s.t.\quad {\mathbf{h}}\left( {{\mathbf{x}}_{i} } \right){{\varvec{\upbeta}}}^{n + 1} = {\mathbf{t}}_{i}^{T} - {{\varvec{\upxi}}}_{i}^{T} ,\,i = 1, \ldots ,N. \\ \end{aligned}$$

By eliminating the constraint conditions and rewriting the Eq. (41) into a matrix form, we can get:

42 $$\ell_{CSRGELM} = - \frac{{{{\varvec{\uptau}}}^{n + 1} }}{{2\sigma^{2} }}\left( {{\mathbf{T}} - {\mathbf{H\upbeta }}^{n + 1} } \right)^{2} + \frac{\lambda }{2}\left\| {{{\varvec{\upbeta}}}^{n + 1} } \right\|_{2,1} + \frac{C}{2}{\text{Tr}} \left( {\left( {{\mathbf{H\upbeta }}^{n + 1} } \right)^{T} {\mathbf{ZH\upbeta }}^{n + 1} } \right).$$

Following the conclusion in [38]. Equation (42) can be rewritten as:

43 $$\begin{aligned} \ell_{CSRGELM} & = - \frac{{{{\varvec{\uptau}}}^{n + 1} }}{{2\sigma^{2} }}\left( {{\mathbf{T}} - {\mathbf{H\upbeta }}^{n + 1} } \right)^{2} + \frac{\lambda }{2}{\text{Tr}} \left( {\left( {{{\varvec{\upbeta}}}^{n + 1} } \right)^{T} {\mathbf{D}}^{n + 1} {{\varvec{\upbeta}}}^{n + 1} } \right) \\ & \quad + \frac{C}{2}{\text{Tr}} \left( {\left( {{\mathbf{H\upbeta }}^{n + 1} } \right)^{T} {\mathbf{ZH\upbeta }}^{n + 1} } \right), \\ \end{aligned}$$

where ${\mathbf{D}}^{n + 1}$ is a diagonal matrix and $d_{ii} = {1 \mathord{\left/ {\vphantom {1 {\left( {2\left\| {{{\varvec{\upbeta}}}_{i}^{n + 1} } \right\|_{2} } \right)}}} \right. \kern-\nulldelimiterspace} {\left( {2\left\| {{{\varvec{\upbeta}}}_{i}^{n + 1} } \right\|_{2} } \right)}}.$ In theory, the value of $\left\| {{{\varvec{\upbeta}}}_{i}^{n + 1} } \right\|_{2}$ can be zero, but this will make the Eq. (43) undifferentiable. To prevent this from happening, a regularization term is added and

44 $$d_{ii}^{{\prime }} = \frac{1}{{2\left( {\sqrt {\left( {{{\varvec{\upbeta}}}_{i}^{n + 1} } \right){{\varvec{\upbeta}}}_{i}^{n + 1} + \kappa } } \right)}},$$

where $\kappa$ is a very small regularization term, in the experiment, $\kappa = 10^{ - 6} .$ It is clear that $d_{ii} = d_{ii}^{^{\prime}}$ when $\kappa \Rightarrow 0.$

Computing the derivative of ${{\varvec{\upbeta}}}^{n + 1}$ about $\ell_{CSRGELM}$ and we have:

45 $$\frac{{\partial \ell_{CSRGELM} }}{{\partial {{\varvec{\upbeta}}}^{n + 1} }} = 0 \Rightarrow - \frac{1}{{\sigma^{2} }}{\mathbf{H}}^{T} {{\varvec{\upomega}}}\left( {{\mathbf{T}} - {\mathbf{H\upbeta }}^{n + 1} } \right) + \lambda {\mathbf{D\upbeta }}^{n + 1} + C{\mathbf{H}}^{T} {\mathbf{ZH\upbeta }}^{n + 1} = 0,$$

where $${{\varvec{\upomega}}} = {\text{diag}} \left( { - {{\varvec{\uptau}}}_{1}^{n + 1} , \ldots , - {{\varvec{\uptau}}}_{N}^{n + 1} } \right).$$

For the case that the number of hidden nodes is less than the number of training samples, the output weights matrix ${{\varvec{\upbeta}}}^{n + 1}$ can be solved as:

46 $$\lambda \sigma^{2} {\mathbf{D}}^{n + 1} {{\varvec{\upbeta}}}^{n + 1} + {\mathbf{H}}^{T} {\mathbf{\omega H\upbeta }}^{n + 1} + C\sigma^{2} {\mathbf{H}}^{T} {\mathbf{ZH\upbeta }}^{n + 1} - {\mathbf{H}}^{T} {\mathbf{\omega T}} = 0,$$

that is

47 $${{\varvec{\upbeta}}}^{n + 1} = \left( {\lambda \sigma^{2} {\mathbf{D}}^{n + 1} + {\mathbf{H}}^{T} {\mathbf{\omega H}} + C\sigma^{2} {\mathbf{H}}^{T} {\mathbf{ZH}}} \right)^{ - 1} {\mathbf{H}}^{T} {\mathbf{\omega T}}.$$

And if the number of hidden nodes is larger than the number of training samples, ${{\varvec{\upbeta}}}^{n + 1}$ may have an unlimited number of solutions. Inspired by the solution of Huang et al. [13],and according to Eq. (46), we make:

48 $$\lambda \sigma^{2} {\mathbf{D}}^{n + 1} {{\varvec{\upbeta}}}^{n + 1} = {\mathbf{H}}^{T} {{\varvec{\upalpha}}} \Rightarrow {{\varvec{\upbeta}}}^{n + 1} = \frac{1}{{\lambda \sigma^{2} }}\left( {{\mathbf{D}}^{n + 1} } \right)^{ - 1} {\mathbf{H}}^{T} {{\varvec{\upalpha}}}.$$

Substituting Eq. (48) into Eq. (46), we have:

49 $${\mathbf{H}}^{T} {{\varvec{\upalpha}}} + \frac{1}{{\lambda \sigma^{2} }}{\mathbf{H}}^{T} {\mathbf{\omega H}}\left( {{\mathbf{D}}^{n + 1} } \right)^{ - 1} {\mathbf{H}}^{T} {{\varvec{\upalpha}}} + \frac{C}{\lambda }{\mathbf{H}}^{T} {\mathbf{ZH}}\left( {{\mathbf{D}}^{n + 1} } \right)^{ - 1} {\mathbf{H}}^{T} {{\varvec{\upalpha}}} - {\mathbf{H}}^{T} {\mathbf{\omega T}} = 0.$$

And multiplying $\left( {{\mathbf{HH}}^{T} } \right)^{ - 1} {\mathbf{H}}$ on both sides of the Eq. (49), we get:

50 $${{\varvec{\upalpha}}} + \frac{1}{{\lambda \sigma^{2} }}{\mathbf{\omega H}}\left( {{\mathbf{D}}^{n + 1} } \right){\mathbf{H}}^{T} {{\varvec{\upalpha}}} + \frac{C}{\lambda }{\mathbf{ZH}}\left( {{\mathbf{D}}^{n + 1} } \right){\mathbf{H}}^{T} {{\varvec{\upalpha}}} - {\mathbf{\omega T}} = 0.$$

Then we obtain the solution of ${{\varvec{\upalpha}}}$:

51 $${{\varvec{\upalpha}}} = \left( {{\mathbf{I}} + \frac{1}{{\lambda \sigma^{2} }}{\mathbf{\omega H}}\left( {{\mathbf{D}}^{n + 1} } \right){\mathbf{H}}^{T} + \frac{C}{\lambda }{\mathbf{ZH}}\left( {{\mathbf{D}}^{n + 1} } \right){\mathbf{H}}^{T} } \right)^{ - 1} {\mathbf{\omega T}}.$$

And ${{\varvec{\upbeta}}}^{n + 1}$ can be computed as:

52 $${{\varvec{\upbeta}}}^{n + 1} = \frac{1}{{\lambda \sigma^{2} }}\left( {{\mathbf{D}}^{n + 1} } \right){\mathbf{H}}^{T} \left( {{\mathbf{I}} + \frac{1}{{\lambda \sigma^{2} }}{\mathbf{\omega H}}\left( {{\mathbf{D}}^{n + 1} } \right){\mathbf{H}}^{T} + \frac{C}{\lambda }{\mathbf{ZH}}\left( {{\mathbf{D}}^{n + 1} } \right){\mathbf{H}}^{T} } \right)^{ - 1} {\mathbf{\omega T}},$$

where ${\mathbf{I}}$ is an identity matrix with dimension of $N$. The analytical solution of ${{\varvec{\upbeta}}}^{n + 1}$ can be finally determined as:

53 $$\left\{ {\begin{array}{*{20}l} {{{\varvec{\upbeta}}}^{n + 1} = \left( {\eta {\mathbf{D}}^{n + 1} + {\mathbf{H}}^{T} {\mathbf{\omega H}} + \rho {\mathbf{H}}^{T} {\mathbf{ZH}}} \right)^{ - 1} {\mathbf{H}}^{T} {\mathbf{\omega T}},} \hfill & {N \ge L} \hfill \\ {{{\varvec{\upbeta}}}^{n + 1} = \frac{1}{\eta }\left( {{\mathbf{D}}^{n + 1} } \right)^{ - 1} {\mathbf{H}}^{T} \left( {{\mathbf{I}} + \frac{1}{\eta }{\mathbf{\omega H}}\left( {{\mathbf{D}}^{n + 1} } \right)^{ - 1} {\mathbf{H}}^{T} + \frac{C}{\lambda }{\mathbf{ZH}}\left( {{\mathbf{D}}^{n + 1} } \right)^{ - 1} {\mathbf{H}}^{T} } \right)^{ - 1} {\mathbf{\omega T}}.} \hfill & {N < L} \hfill \\ \end{array} } \right.$$

And $\eta = \lambda \sigma^{2} , \, \rho = C\sigma^{2} .$ It is worth noting that ${{\varvec{\upbeta}}}^{n + 1}$ is a dependence on ${\mathbf{D}}^{n + 1} ,$ so an iterative optimization algorithm is proposed for solving ${{\varvec{\upbeta}}}^{n + 1}$ and ${\mathbf{D}}^{n + 1} .$ The flow of Algorithm 1 is as follows:
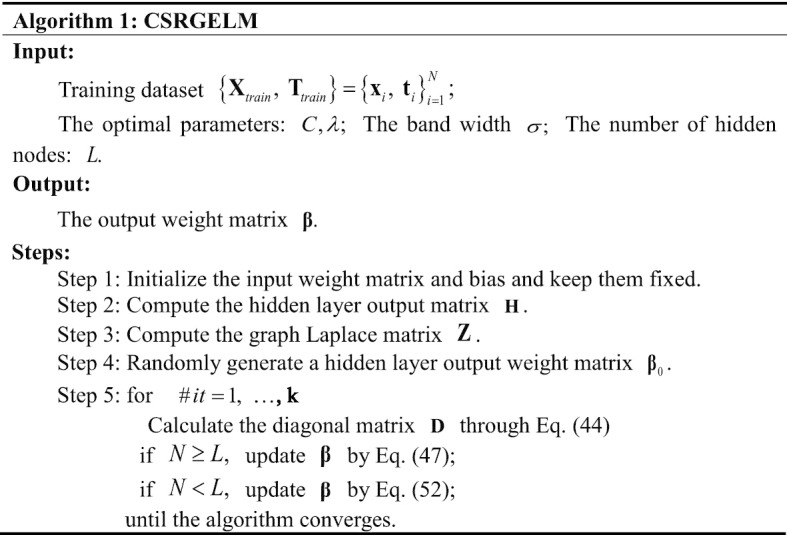


#### Computational complexity analysis

The computational complexity of CSRGELM is analyzed in this subsection. We define $M$ as the number of classes. In Eq. (47), we have to calculate ${\mathbf{D}}^{n + 1} ,$ ${\mathbf{H}}^{T} {\mathbf{\omega H}},$ ${\mathbf{H}}^{T} {\mathbf{ZH}},$ ${\mathbf{H}}^{T} {\mathbf{\omega T}},$ ${{\varvec{\upomega}}}$ and $\left( {\lambda \sigma^{2} {\mathbf{D}}^{n + 1} + {\mathbf{H}}^{T} {\mathbf{\omega H}} + C\sigma^{2} {\mathbf{H}}^{T} {\mathbf{ZH}}} \right)^{ - 1} .$ The computational cost for ${\mathbf{D}}^{n + 1}$ is $O\left( {LM} \right),$ and it needs $O\left( {L^{2} N} \right)$ to compute ${\mathbf{H}}^{T} {\mathbf{\omega H}}$ and ${\mathbf{H}}^{T} {\mathbf{ZH}}.$ For ${\mathbf{H}}^{T} {\mathbf{\omega T}},$ the computational complexity is $O\left( {LNM} \right),$ and the computational complexity for $\left( {\lambda \sigma^{2} {\mathbf{D}}^{n + 1} + {\mathbf{H}}^{T} {\mathbf{\omega H}} + C\sigma^{2} {\mathbf{H}}^{T} {\mathbf{ZH}}} \right)^{ - 1}$ is $O\left( {L^{3} } \right),$ while it needs $O\left( {NM} \right)$ to compute ${{\varvec{\upomega}}}.$ In addition, the computational time complexity of the operation of $\left( {\lambda \sigma^{2} {\mathbf{D}}^{n + 1} + {\mathbf{H}}^{T} {\mathbf{\omega H}} + C\sigma^{2} {\mathbf{H}}^{T} {\mathbf{ZH}}} \right)^{ - 1}$ multiplied by ${\mathbf{H}}^{T} {\mathbf{\omega T}}$ is $O\left( {L^{2} M} \right).$ Owing to $N > L,$ The computational cost of Eq. (47) is $O\left( {L^{2} N} \right).$ Assuming that the method converges after $K$ iterations, we can obtain that the final computational cost of CSRGELM is $K \times O\left( {L^{2} N} \right).$

#### Robustness analysis

**Fig. 9:**
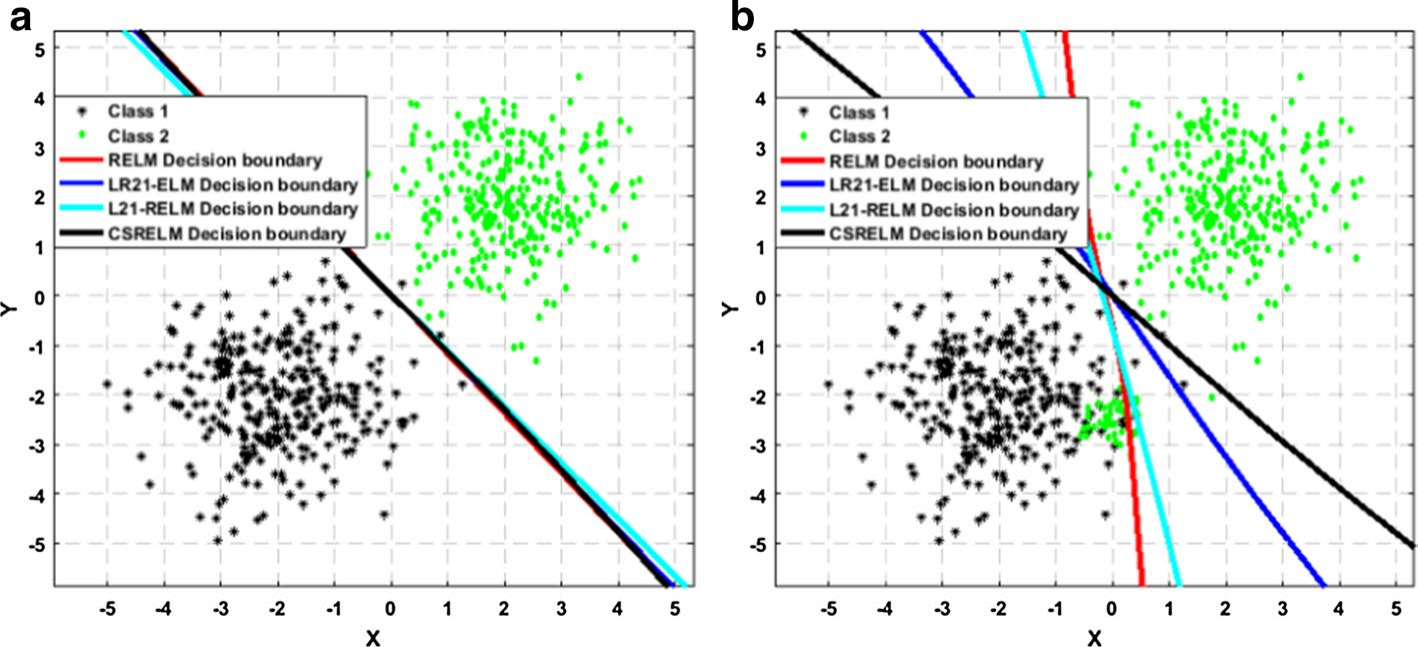
Classification decision boundary on artificial Gaussian dataset with 300 samples and 50 noise

**Fig. 10:**
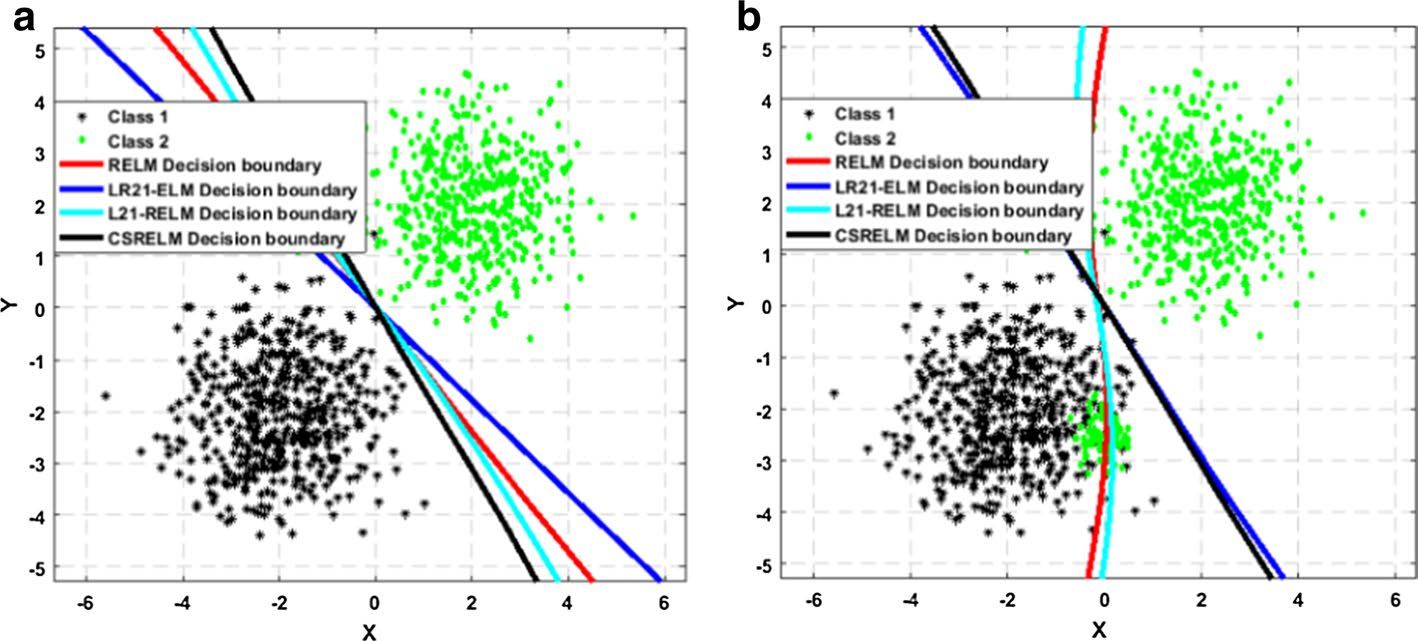
Classification decision boundary on artificial Gaussian dataset with 500 samples and 100 noise

An experiment is designed to demonstrate the robustness of CSRGELM to outliers and noise. Two groups of data subject to Gaussian distribution that are randomly generated. Class 1 includes 300 samples with mean parameter $\chi_{1} = \left[ { - 2, - 2} \right]$ and covariance matrix $\phi_{1} = \left[ {1 0; 0 1} \right],$ while class 2 includes another 300 samples with mean parameter $\chi_{2} = \left[ {2, 2} \right]$ and covariance matrix $\phi_{2} = \left[ {1 0; 0 1} \right].$ And in the experiments, RELM, *L*_2,1_-RFELM, LR21ELM and CSRGELM are trained on this dataset, respectively. The classification decision boundary has shown in Fig. 9. Figure 9a is the classification results with no noise, and it shows that these two classes are separated easily. Figure 9b is the classification results with 50 noise, these noisy points originally belong to the class 2 but are confused in the class 1. And Fig. 9b shows that under the interference of noise, the classification decision boundaries of these four methods have changed. And the changes of RELM and *L*_2,1_-RFELM are more obvious. Again, another dataset is generated, class 1 and class 2 have 500 samples, respectively. First, four methods are trained on this dataset and the classification decision boundary is shown in Fig. 10a. It is obvious that the data can be separated by four straight lines. And then, 100 points belonging to class 2 are confused into class 1 as the noise. The final classification results have been shown in Fig. 10b. Clearly, RELM and *L*_2,1_-RFELM try to fit the noise, and their classification decision boundaries are already unreliable. But due to the constraints of the robust loss function, the classification decision boundaries of CSRGELM and LR21ELM are hardly affected.

## Data Availability

The TCGA datasets that support the findings of this study are available in https://www.cancer.gov/about-nci/organization/ccg/research/structural-genomics/tcga. The UCI datasets that support the findings of this study are available in https://archive.ics.uci.edu/ml/datasets.php. The datasets of g50c, COIL20, and USPST that support the findings of this study are included in this published article [13].

## Abbreviations

ELM: Extreme learning machine
CSRGELM: Correntropy induced loss based sparse robust graph regularized extreme learning machine
SLFNs: Single hidden layer feedforward neural network
CIM: Correntropy induced metric
CH-loss: Correntropy loss and hinge loss
CHELM: Extreme learning machine with the CH-loss
TCGA: The Cancer Genome Atlas
UCI: University of California Irvine
Acc: Accuracy
Pre: Precision
F-mea: F-measure
